# SIRT7-mediated deacetylation of XRCC6 at lysine 591 drives breast cancer progression

**DOI:** 10.3389/fonc.2026.1806267

**Published:** 2026-05-08

**Authors:** Jie Yin, Jiaxing Liu, Meirui He, Tang Xiao, Wenjuan Xiang, Ruxia Sheng, Haifang Zhao, Xinyue Huang, Sheng Xu, Yong Zhou, Junfeng Liu, Ruijuan Heng, Xiaoying He, Yunxiang Wang, Yu Song, Pan Qi

**Affiliations:** 1 The Fourth Clinical College, Henan Medical University, Xinxiang, Henan, China; 2 College of Pharmacy, Henan Medical University, Xinxiang, Henan, China; 3 Department of Head and Neck Breast, Xinxiang Central Hospital, the Fourth Affiliated Hospital of Henan Medical College, Xinxiang, China

**Keywords:** acetylation modification, breast cancer, PARylation modification, poly [ADP-ribose] polymerase 1, X-ray repair cross-complementing protein 6

## Abstract

**Rationale:**

The acetylation level of XRCC6 is significantly decreased in clinical breast cancer tissues compared with paracancerous tissues; however, the relationship between this alteration and the occurrence and development of breast cancer remains unclear.

**Methods:**

Acetylproteomics was employed to detect changes in protein acetylation levels in clinical samples. Co-immunoprecipitation (Co-IP) assays were utilized to identify the acetyltransferase and deacetylase responsible for XRCC6 K591 acetylation. Transwell chamber assays were performed to evaluate the impact of XRCC6 K591 acetylation on the invasive capability of breast cancer cells, while colony formation assays were conducted to assess its effect on clonogenicity.

**Results:**

EP300 was identified as the acetyltransferase responsible for catalyzing XRCC6 acetylation, while Sirt7 functions as its specific deacetylase. Downregulation of XRCC6 acetylation at K591 promotes its interaction with PARP1, thereby elevating XRCC6 PARylation levels and augmenting the DNA damage repair capacity of breast cancer cells. Furthermore, *in vivo* experiments demonstrated that reduced XRCC6 acetylation promotes tumor growth and increases tumor volume in xenograft models.

**Conclusion:**

Our findings elucidate a novel mechanism by which downregulated XRCC6 acetylation drives breast cancer progression.

## 1 Introduction

Breast cancer is a common clinical malignancy with an incidence rate that is gradually increasing. According to the World Health Organization (WHO), the incidence of breast cancer has surpassed that of lung cancer and colon cancer, ranking first among malignancies in Chinese women (1, 2). Although factors such as abnormal hormone levels, alterations in the tumor microenvironment, and aberrant cellular DNA damage repair are closely related to the occurrence and development of breast cancer, its pathogenesis remains unclear (3). Elucidating the mechanism of breast cancer pathogenesis is the foundation for targeted therapy. Post-translational modifications (PTMs) of proteins play a critical role in tumorigenesis and development (4, 5). In particular, acetylation can regulate the stability or function of various key proteins in tumor cells, thereby influencing tumor initiation and metastasis (6, 7). For example, acetylation at specific histone sites regulates the expression levels of breast cancer-related genes (8, 9).Our previous studies have also found that acetylation of certain non-histone proteins significantly impacts tumorigenesis in colon cancer (10). Therefore, this study investigated the acetylation status of non-histone proteins in breast cancer. The results indicate that the acetylation level of X-ray Repair Cross-Complementing Protein 6(XRCC6) is downregulated in breast cancer.

XRCC6, also commonly known as Ku70, is a crucial DNA repair protein within the cell (11). Its classic function is participation in the NHEJ (Non-Homologous End Joining) repair pathway (12). When a DNA double-strand break occurs, XRCC6 rapidly forms a Ku heterodimer with X-ray Repair Cross-Complementing Protein 5, or Ku80(XRCC5) (13–15). This complex recognize and tightly bind to the broken DNA ends. This not only protects the broken ends from degradation by nucleases but, more importantly, serves as a platform to recruit other key repair proteins to assemble the DNA-PK(DNA-dependent Protein Kinase Catalytic Subunit) complex (16, 17).

Beyond its core role in NHEJ, XRCC6 participates in other cellular activities to maintain genomic stability (18, 19). Studies suggest that XRCC6 may regulate homologous recombination repair pathways during the S and G2 phases of the cell cycle by interacting with key homologous recombination proteins such as Breast Cancer Type 1 Susceptibility Protein(BRCA1) and RAD51 Recombinase(RAD51), assisting cells in selecting the most appropriate repair mechanism based on the damage type and cell cycle stage (20, 21). Aberrant function or expression of XRCC6 leads to the accumulation of DNA damage and increased genomic instability, thereby significantly elevating the risk of cellular transformation (22). Abnormally high expression of XRCC6 has been observed in various cancers (23). And its overexpression correlates with enhanced cancer cell proliferation and poor patient prognosis in osteosarcoma (24). Furthermore, polymorphisms in the XRCC6 gene may influence individual susceptibility to diseases such as colorectal cancer (25).

Our results indicate that the acetylation level at lysine 591 (K591) of XRCC6 is significantly reduced in breast cancer. This downregulation significantly promotes breast cancer cell growth by modulating the function of factors related to cellular DNA damage repair.

## 2 Results

### 2.1 XRCC6 acetylation levels are significantly downregulated in breast cancer

**Figure 1:**
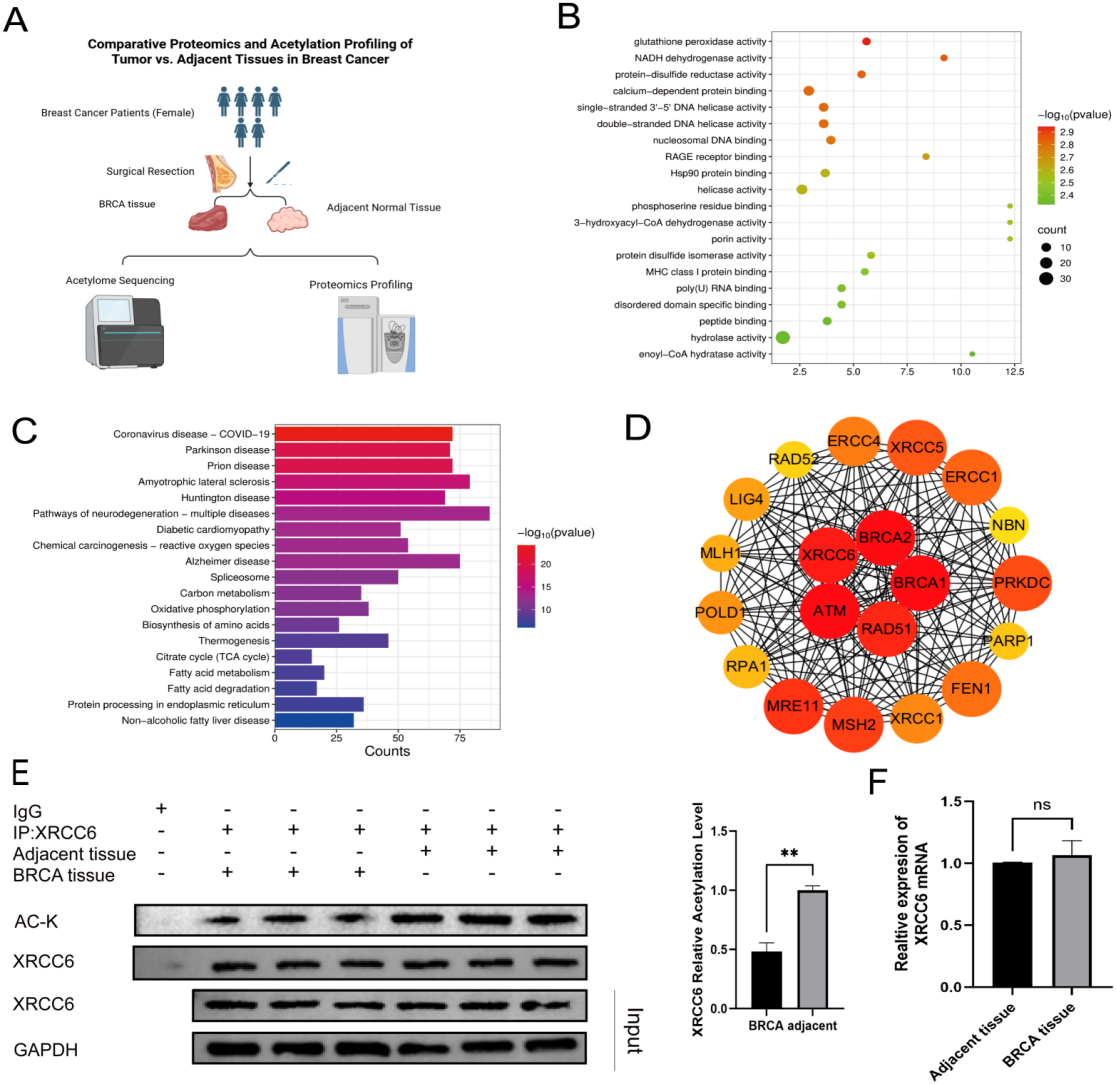
XRCC6 acetylation levels are significantly downregulated in breast cancer tissues compared to adjacent non-cancerous tissues. **(A)** Schematic workflow for the proteomic and acetylomic analyses of breast cancer and adjacent non-cancerous tissue samples. **(B, C)** GO and KEGG analyses of the top 500 proteins with the most significant acetylation downregulation. **(D)** Protein-Protein Interaction analysis of the top 20 proteins with the most significant decrease in acetylation modification. **(E)** Immunoprecipitation assays detected the acetylation level of XRCC6 in breast cancer and para-carcinoma tissues using(n=3). **(F)** RT-PCR detected the XRCC6 mRNA expression levels (n=3). Data are presented as the mean ± SD, Student’s t-test was used. ns = not significant, *P < 0.05, **P < 0.01.

Previous research in our laboratory showed significant differences in the expression levels of acetyltransferases related to protein acetylation, such as N-Acetyltransferase 10 (NAT10) or E1A Binding Protein p300(EP300), between breast cancer tissues and adjacent non-cancerous tissues (10). Based on this, we hypothesized that protein acetylation levels might differ significantly between these tissues. We collected 10 pairs of breast cancer and para-carcinoma tissues and performed proteomics and acetylomics analyses (**Figure 1A**). Compared to para-carcinoma tissues, the number of proteins with downregulated acetylation levels in breast cancer tissues exceeded those with upregulated levels. GO and KEGG analyses of the top 500 proteins with the most significant downregulation are shown in **Figures 1B, C**. Protein-Protein Interaction analysis of the top 20 proteins with the most significant decrease in acetylation modification identified XRCC6 as one of the most important proteins (**Figure 1D**). Specifically, the acetylation level at the K591 of XRCC6 in breast cancer dropped to one-quarter of that in para-carcinoma tissues. Considering the critical role of XRCC6 in the development of various tumors, we validated the acetylation level of XRCC6 in breast cancer and para-carcinoma tissues using immunoprecipitation assays. Consistent with the mass spectrometry results, the acetylation level of XRCC6 was significantly reduced in breast cancer tissues (**Figure 1E**), while there was no significant difference in XRCC6 mRNA expression levels (**Figure 1F**). This suggests that the downregulation of XRCC6 acetylation plays an important role in breast cancer.

### 2.2 Sirt7 is the deacetylase of XRCC6, while EP300 is its acetylase

**Figure 2:**
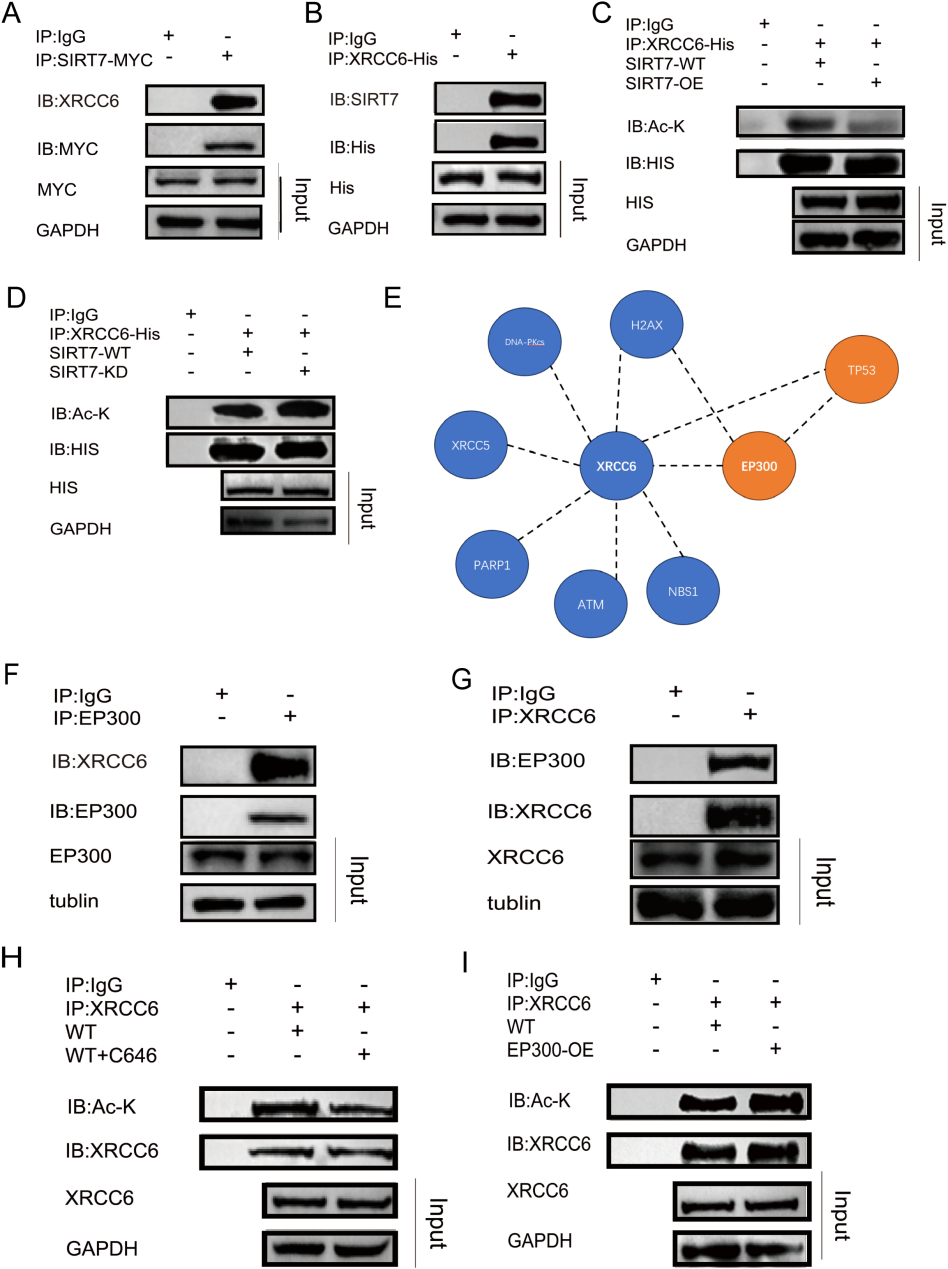
The deacetylase and acetylase of XRCC6. **(A)** Immunoprecipitation using a Sirt7 antibody improved XRCC6 interacted with Sirt7 in MCF-7 cell. **(B)** Immunoprecipitation using a XRCC6 antibody improved Sirt7 interacted with XRCC6. **(C)** Overexpression of Sirt7 significantly reduced XRCC6 acetylation levels. **(D)** knockdown of Sirt7 increased XRCC6 acetylation levels. **(E)** protein interaction databases indicated that EP300 could interact with XRCC6. **(F, G)** Co-immunoprecipitation experiments confirmed that EP300 binds to XRCC6. **(H)** EP300 inhibitor C646 reduced XRCC6 acetylation levels. **(I)** EP300 overexpression increased XRCC6 acetylation levels.

To investigate the enzymes catalyzing the acetylation and deacetylation of XRCC6, we first captured all proteins interacting with XRCC6 via IP experiments and performed mass spectrometry. The results identified Sirtuin 7(Sirt7) as an XRCC6-binding protein(**Supplementary Figure 1A**). Co-immunoprecipitation using a Sirt7 antibody detected XRCC6 among the interacting proteins in MCF-7 cell line (**Figure 2A**). Correspondingly, Sirt7 was found among XRCC6-binding proteins, leading us to hypothesize that Sirt7 is the deacetylase for XRCC6 (**Figure 2B**). Further experiments showed that overexpression of Sirt7 significantly reduced XRCC6 acetylation levels, while knockdown of Sirt7 increased them (**Figures 2C, D**). These results confirm Sirt7 as the deacetylase of XRCC6. To identify the acetylase for XRCC6, we searched several protein interaction databases (UniProt, STRING, and BioGRID), which indicated that EP300 could interact with XRCC6 (**Figure 2E**). Co-IP experiments confirmed that EP300 binds to XRCC6 (**Figures 2F, G**). Furthermore, the use of the EP300 inhibitor C646 reduced XRCC6 acetylation levels (**Figure 2H**), while EP300 overexpression increased them (**Figure 2I**). The results prove that EP300 is the acetylase for XRCC6.

### 2.3 Downregulation of XRCC6 K591 acetylation promotes breast cancer development

**Figure 3:**
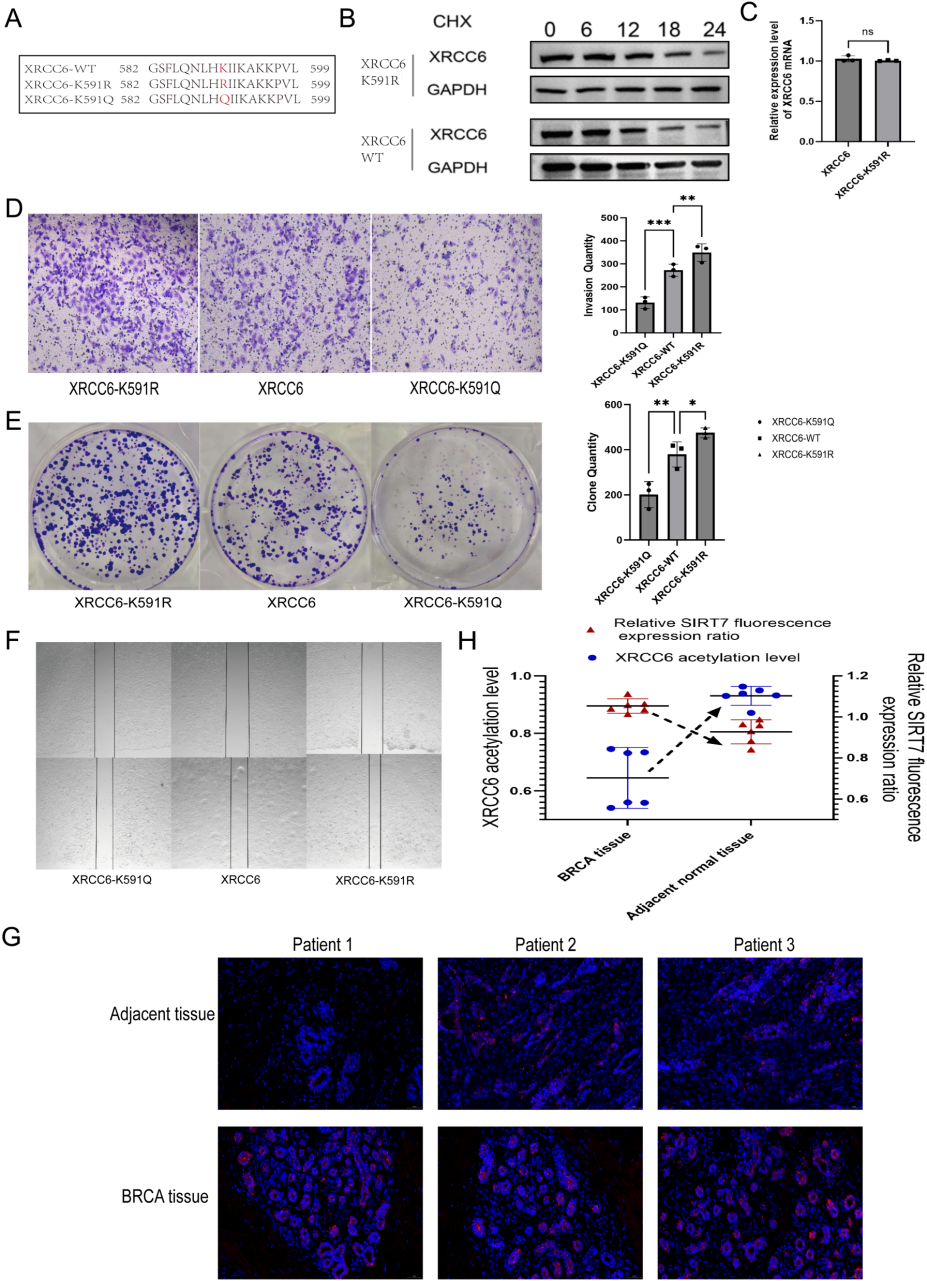
Downregulation of XRCC6 acetylation at the K591 site promotes breast cancer. **(A)** An acetylation-mimetic mutant (K591Q) and a deacetylation-mimetic mutant (K591R) were constructed. **(B)** The acetylation modification at K591 did not significantly affect the half-life of the XRCC6 protein. **(C)** The K591R mutation did not influence XRCC6 mRNA expression levels. **(D-F)** Transwell, colony formation, and wound healing assays demonstrated the non-acetylatable K591R mutant enhanced the invasion, colony formation, and migration capabilities of MCF-7 cells, whereas the acetylation-mimetic K591Q mutant displayed the opposite effect. **(G)** Immunofluorescence assays demonstrated that Sirt7 expression was significantly higher in clinical breast cancer tissues compared to para-carcinoma tissues. **(H)** Sirt7 fluorescence intensity was inversely proportional to relative XRCC6 acetylation levels(n=6).

To analyze the effect of acetylation on XRCC6 function, we examined the protein mass spectrometry data. Results showed that acetylation at the K591 site of XRCC6 was significantly lower in breast cancer samples compared to para-carcinoma tissues (**Supplementary Figure 1B**). We constructed an acetylation-mimetic mutant (K591Q) and a deacetylation-mimetic mutant (K591R) to assess differences in protein stability (**Figure 3A**). Results indicated that acetylation modification at this site did not significantly affect the half-life of the XRCC6 protein, nor did the K591R mutation alter XRCC6 mRNA expression levels (**Figures 3B, C**).

We observed the effects of acetylation at this site on cell colony formation, invasion, and migration. The results indicated that compared to the wild-type XRCC6, the non-acetylatable K591R mutant significantly enhanced the invasion, colony formation, and migration capabilities of MCF-7 and T47D breast cancer cells. Conversely, acetylation-mimetic K591Q inhibited proliferation and invasion (**Figures 3D-F**; **Supplementary Figures 1 C, D**). This is consistent with the significant decrease in XRCC6 acetylation detected in clinical breast cancer samples. We also observed that Sirt7 expression was significantly higher in clinical breast cancer tissues compared to para-carcinoma tissues (**Figure 3G**), and Sirt7 fluorescence intensity was inversely proportional to relative XRCC6 acetylation levels (**Figure 3H**). This suggests that in breast cancer, high expression of Sirt7 may influence tumor development by lowering XRCC6 acetylation levels.

### 2.4 Downregulation of XRCC6 acetylation enhances its interaction with PARP1

**Figure 4:**
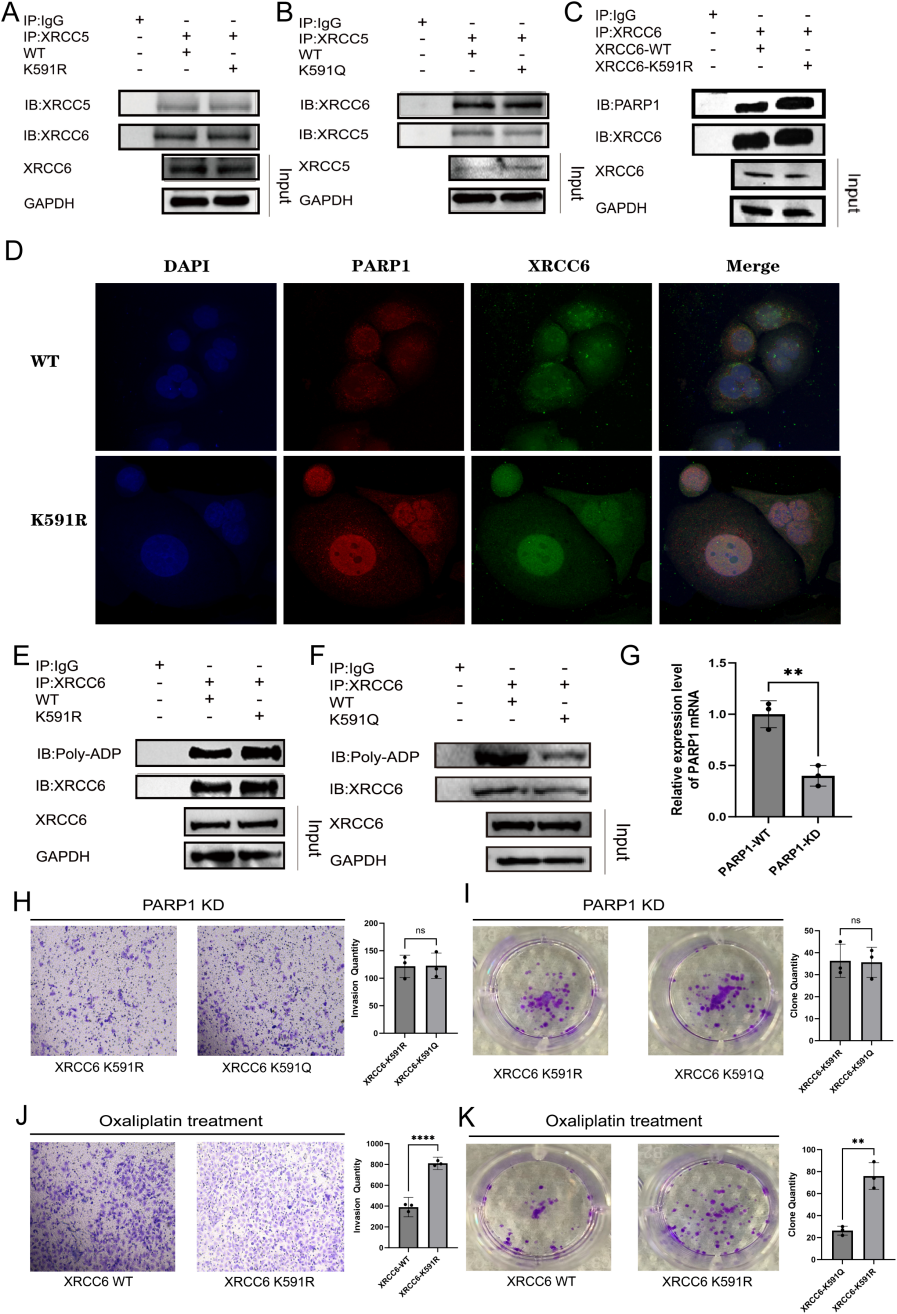
Downregulation of XRCC6 acetylation promoted its interaction with PARP1. **(A, B)** Immunoprecipitation assay indicated that neither K591R nor K591Q affected the interaction between XRCC6 and XRCC5. **(C)** Immunoprecipitation assay demonstrated K591R significantly enhanced the binding of XRCC6 to PARP1. **(D)** Immunofluorescence co-localization assays verified K591R facilitate the binding of XRCC6 to PARP1. **E** and **(F)** K591R enhanced XRCC6 self-PARylation. Conversely, the K591Q mutation reduced XRCC6 self-PARylation. **(G)** the PARP1 mRNA expression levels in WT and PARP1 knockdown MCF-7 cell. **(H, I)**, Transwell and colony formation assays demonstrated the effect of K591R or K591Q mutations on cell invasion and colony formation were greatly reduced(n=3). **(J, K)** Transwell and colony formation assays indicated that under the pressure of DNA-damaging agents, the XRCC6 K591R mutant significantly promoted cell survival and invasion(n=3). Data are presented as the mean ± SD, Student’s t-test was used. ns = not significant, *P < 0.05, **P < 0.01,****P < 0.0001.

XRCC6 often forms a heterodimer with XRCC5 during DNA damage repair, and XRCC6 can be catalyzed by Poly [ADP-Ribose] Polymerase 1 (PARP1) to undergo poly-ADP-ribosylation (PARylation) modification (26, 27). This complex plays an important role in DNA damage repair. Therefore, we hypothesized that the downregulation of XRCC6 acetylation might affect its binding to XRCC5. However, results showed that neither K591R nor K591Q affected the interaction between XRCC6 and XRCC5 (**Figures 4A, B**). Interestingly, the K591R significantly enhanced the binding of XRCC6 to PARP1 (**Figure 4C**), which was confirmed by immunofluorescence co-localization assays (**Figure 4D**). Considering that PARP1 catalyzes the PARylation of downstream proteins to promote DNA repair, we examined the effect of K591R on XRCC6’s self-PARylation levels. Results confirmed that K591R enhanced XRCC6 self-PARylation by promoting its interaction with PARP1 (**Figure 4E**). Conversely, the K591Q mutation reduced XRCC6 self-PARylation levels (**Figure 4F**).

Based on these results, we infer that the decrease in acetylation at the K591 of XRCC6 enhances its binding to PARP1, thereby promoting XRCC6 self-PARylation and the DNA damage repair process. Consequently, we knocked down PARP1 expression (**Figure 4G**). Results showed that the effects of K591R or K591Q mutations on breast cancer cell invasion and colony formation were greatly reduced (**Figures 4H, I**). These data proved that the downregulation of XRCC6 K591 acetylation may enhance breast cancer cell proliferation by augmenting DNA damage repair functions.

Given the important role of PARP1-mediated PARylation of XRCC6 in DNA damage and tumor drug resistance (28, 29), we treated MCF-7 cells with low-dose oxaliplatin. Results showed that under the pressure of DNA-damaging agents, the XRCC6 K591R mutant significantly promoted cell survival and invasion (**Figures 4J,K**). This indicates that the downregulation of XRCC6 K591 acetylation indeed promotes tumor cell survival via DNA damage pathways.

### 2.5 Downregulation of XRCC6 K591 acetylation promotes breast cancer growth in a xenograft model

**Figure 5:**
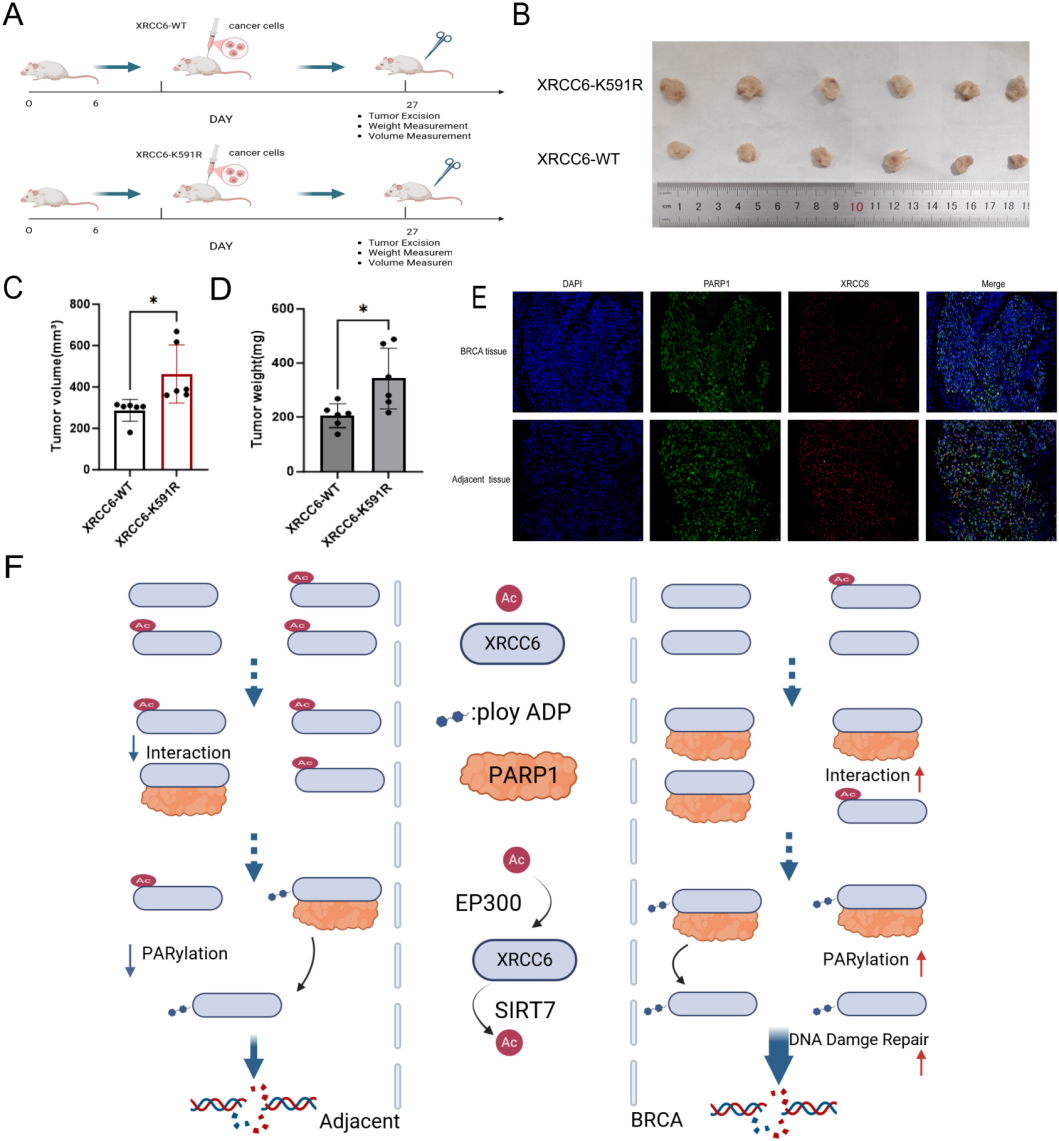
Downregulation of XRCC6 K591 acetylation promotes breast cancer growth in a xenograft model. **(A)**. Experimental workflow for the generation of MCF-7 cells stably expressing wild-type or K591R mutant XRCC6 and the subsequent nude mouse xenograft assay. **(B-D)**. Comparison of tumor size, volume(*P* = 0.0166), and weight(*P* = 0.0194) of subcutaneous xenografts derived from MCF-7 cells stably expressing wild-type (WT) or K591R mutant XRCC6(n=6). **(E)**. Dual immunofluorescence labeling of XRCC6 and PARP1 indicated that co-localization of XRCC6 and PARP1 was higher in clinical breast cancer tissues compared to para-carcinoma tisssues. **(F)** Molecular mechanism by which downregulation of XRCC6 K591 acetylation promotes breast cancer development. Data are presented as the mean ± SD, Student’s t-test was used. ns = not significant, *P < 0.05.

To observe the effect of XRCC6 K591 acetylation downregulation on breast cancer growth *in vivo*, we introduced the K591R mutant XRCC6 into MCF-7 cells via lentivirus to construct stable cell lines. Wild-type and K591R mutant breast cancer cells were transplanted subcutaneously into nude mice. Tumors were harvested after three weeks (**Figure 5A**). Results showed that tumors generated by K591R mutant cells were larger in volume(*p* = 0.0169) and heavier(*p* = 0.0194) than those from wild-type cells (**Figures 5B-D**). This indicates that the decrease in K591 acetylation promotes tumor development.

In clinical breast cancer samples, dual immunofluorescence labeling of XRCC6 and PARP1 revealed relatively high XRCC6 content in breast cancer tissues. Furthermore, co-localization of XRCC6 and PARP1 was higher in tumor tissues compared to para-carcinoma tissue. This is consistent with cellular experimental results, indicating that deacetylated XRCC6 interacts more with PARP1 and participates in DNA damage repair (**Figure 5E**). Summarizing all experimental results, we conclude that Sirt7-induced downregulation of acetylation at the K591 site of XRCC6 significantly promotes the binding of XRCC6 and PARP1, thereby enhancing XRCC6 PARylation and DNA damage repair (**Figure 5F**).

## 3 Discussion

The occurrence of breast cancer is multifactorial, with studies revealing the influence of genetic factors, hormonal changes, and immunological alterations (30, 31). Research on the role of acetylation in breast cancer pathogenesis remains limited and has focused primarily on histone acetylation (32, 33). However, investigations of acetylation levels in clinical samples reveal significant changes in the acetylation of many non-histone proteins (34, 35).

This study found that the acetylation level of the DNA damage repair protein XRCC6 is significantly downregulated in breast cancer. Our results show that EP300 is the acetylase for XRCC6, while Sirt7 is its deacetylase. The acetylation site of XRCC6 is at K591, but deacetylation at this site does not affect XRCC6 protein stability. However, the deacetylation-mimetic K591R mutation significantly enhanced the invasion, proliferation, and migration capabilities of breast cancer cells, whereas the acetylation-mimetic K591Q exerted the opposite effect. This indicates that XRCC6 plays an important role in the development of breast cancer. To explore why XRCC6 K591 acetylation is significantly reduced in breast cancer, we found that the expression of its deacetylase, Sirt7, is significantly upregulated in breast cancer, and Sirt7 levels are inversely proportional to XRCC6 acetylation levels. This proves that the decrease in XRCC6 acetylation is likely induced by Sirt7. The expression levels of Sirt7 in primary breast cancer tissues and metastatic lesions are significantly correlated with metastasis-free survival in patients (36, 37). Current literature suggests that Sirt7 may exert dual roles, functioning as both an oncogene and a tumor suppressor within the context of breast cancer (36, 38). Specifically, several studies have demonstrated that Sirt7 facilitates the suppression of E-cadherin expression through epigenetic regulation, thereby reducing intercellular adhesion (39, 40). Furthermore, it also enhances TGF-β signaling, which promotes the acquisition of mesenchymal characteristics (36). Conversely, our experimental data reveal that Sirt7 also modulates DNA damage repair capacity in breast cancer cells by regulating the acetylation status of XRCC6.

Further mechanistic studies found that the decrease in XRCC6 acetylation promotes its interaction with PARP1. This interaction leads to a significant increase in XRCC6 PARylation levels. PARylation of XRCC6 aids its rapid localization to break sites during DNA double-strand breaks and facilitates the recruitment of downstream repair protein complexes (such as DNA-PKcs). Therefore, elevated PARylation of XRCC6 significantly enhances its DNA damage repair capacity, promoting breast cancer cell survival. This study also found that in the presence of oxaliplatin, the K591R mutation significantly increased the colony formation ability of MCF-7 cells.

Consistent with cellular experiments, we observed in a murine breast cancer xenograft model that the acetylation-deficient mimic XRCC6 K591R significantly promoted the weight and volume of xenografted breast tumors. Furthermore, in clinical breast cancer tissue sections, XRCC6 acetylation levels were observed to be significantly lower than in para-carcinoma tissues, while XRCC6 and PARP1 co-localization was more frequent. The DNA damage repair (DDR) function of tumor cells differs significantly from that of normal cells, representing a critical target for antineoplastic drug development (41). Historically, research strategies have predominantly focused on the direct inhibition of key proteins within this pathway (42). However, our experimental results demonstrate that modulating the post-translational modification (PTM) levels of these proteins offers an alternative approach to regulate DDR function in tumor cells. We aim to develop small-molecule compounds that disrupt the interaction between XRCC6 and Sirt7, and to evaluate their druggability and therapeutic potential against breast cancer.

In conclusion, this study elucidates the relationship between the downregulation of XRCC6 K591 acetylation and breast cancer, identifying Sirt7 as the primary enzyme catalyzing this deacetylation. It also provides a new strategy for breast cancer drug development: future efforts could focus on developing inhibitors targeting the XRCC6 K591 site to prevent its binding with the deacetylase Sirt7, thereby maintaining its acetylation level and inhibiting breast cancer progression. Naturally, this study has limitations, such as the insufficient sample size of clinical specimens, and further in-depth research is required.

## 4 Materials and Methods

### 4.1 Cell culture and drug treatment

MCF-7 cells were routinely cultured in DMEM complete medium supplemented with 10% fetal bovine serum (FBS) and 1% penicillin-streptomycin. Cells were maintained in a humidified incubator at 37 C with 5% CO_2_. Cells were passaged every 2–3 days. Cells in the logarithmic growth phase were used for experiments.

The complete list of drugs used in this experiment is provided in **Supplementary data Table 3**.

### 4.2 Plasmid transfection

MCF-7 cells in the logarithmic growth phase were seeded into 6-well plates at a density of 3 × 10^^5^ cells per well. For Lentiviral Packaging: When cell confluence reached 50%–60%, pMD2G, psPAX2, and the target plasmid(**Supplementary data Table 2**) were mixed into Opti-MEM Reduced Serum Medium. The mixture was combined with Lipofectamine 3000 reagent and transfected into 293T cells. After 12 hours, the medium on the 293T cells was replaced with fresh medium. After 2 days, the supernatant containing lentiviral particles was collected and added to the culture medium of the target cells.

For Transient Transfection: Plasmids(**Supplementary data Table 2**) and Lipofectamine 2000 Transfection Reagent were added to serum-free medium in the appropriate proportions. The mixture was incubated at room temperature for 20 minutes and then added to the culture dishes of the target cells. Transfection efficiency was assessed 48 hours post-transfection.

### 4.3 Co-immunoprecipitation

Transfected cells were lysed. 50 μL of magnetic beads were placed in a 1.5 mL centrifuge tube and positioned on a magnetic rack. After the beads settled, the supernatant was removed. Diluted antibodies(**Supplementary data Table 4**) were added and incubated with the beads with rotation for 1 hour. The tube was placed back on the magnetic rack, the supernatant was removed, and the beads were washed 3 times with 200 μL of TBST. Protein samples were then added and incubated for 1 hour, followed by 3 washes with TBST. The complex was resuspended in 20 μL of Elution Buffer and 5 μL of $5\times$ SDS-PAGE loading buffer. The samples were boiled at 95–100 °C for 8 minutes, and the supernatant was collected for subsequent experiments.

### 4.4 Western blot

Cell protein samples were separated by SDS-PAGE and electro transferred onto PVDF membranes. Membranes were blocked with 5% non-fat milk at room temperature for 2 hours. After washing 3 times with TBST, membranes were incubated with specific primary antibodies(**Supplementary data Table 4**) overnight at 4 °C. After 3 washes with TBST, membranes were incubated with secondary antibodies at room temperature for 1.5 hours. Following additional washes, bands were visualized using an ECL chemiluminescent substrate and captured using an imaging system.

### 4.5 Protein stability assay

Cells from each group were seeded into 6-well plates. Upon reaching 80% confluence, cycloheximide (CHX) was added to the medium to inhibit *de novo* protein synthesis. Cells were harvested at 0, 6, 12, 18, and 24 hours post-CHX treatment. Total protein was extracted for Western blot analysis to monitor the degradation of the XRCC6 protein over time.

### 4.6 Transwell invasion assay

Log-phase cells were digested with 0.25% trypsin, centrifuged, and resuspended in serum-free medium to a density of $2.5 \times 10^5$ cells/mL. Transwell inserts were placed in 24-well plates. The lower chamber was filled with 500 μL of DMEM containing 20% FBS, while 200 μL of the cell suspension was added to the upper chamber. Cells were incubated at 37 C in a 5% CO_2_ incubator for 24 hours. The inserts were removed, the medium was discarded, and cells were fixed with 4% paraformaldehyde (PFA) for 30 minutes. After washing with distilled water for 2 minutes, cells were stained with crystal violet for 10 minutes. Following 2–3 washes with PBS, cells were observed and imaged under a microscope.

### 4.7 Colony formation assay

Log-phase cells from each group were digested, resuspended, and counted. Cells were seeded into 6-well plates at a density of 500 cells per well, with 2 mL of complete medium added to each well. Plates were incubated at 37 C with 5% CO$_2$ for 10–14 days, with fresh medium replacement every 3 days. After the culture period, the medium was discarded, and cells were washed twice with PBS. Cells were fixed with 1 mL of 4% PFA per well for 15 minutes. After washing with PBS, cells were stained with 0.5 mL of 0.1% crystal violet solution per well for 15 minutes. The staining solution was discarded, and plates were gently washed twice with PBS and air-dried at room temperature. Each well was photographed.

### 4.8 Wound healing assay

Log-phase cells from each group were digested, counted, and seeded into 24-well plates at a density of $1 \times 10^5$ cells per well. Cells were cultured in complete medium until reaching approximately 80% confluence to form a uniform monolayer. A linear scratch was created in the center of the cell monolayer using a sterile 20 μL pipette tip (held vertically). The medium was discarded, and cells were gently washed twice with PBS to remove detached cells, followed by medium replacement. Images of the same scratch position were captured under a microscope at 0 and 24 hours post-scratching.

### 4.9 Real-time quantitative PCR

Total RNA was extracted following the manufacturer’s protocol. Subsequently, reverse transcription was performed to synthesize cDNA. Quantitative PCR (qPCR) was conducted in a 20 µL reaction volume using a SYBR Green system. Relative mRNA expression levels were calculated using the 2^-△△Ct^ method. The primer sequences are listed in **Supplementary Table 6**.

### 4.10 Bioinformatics analysis

This study utilized public bioinformatics databases and tools to predict the sequence characteristics, evolutionary conservation, and protein-protein interaction (PPI) networks of XRCC6, providing a theoretical basis for subsequent experiments. All analyses were based on the latest available versions of the databases.

Evolutionary Conservation Analysis: To assess the evolutionary significance of the target acetylation site (K591), orthologous sequences of human XRCC6 in various vertebrates (e.g., Homo sapiens, Mus musculus, Rattus norvegicus, Danio rerio) were extracted from UniProt. Multiple sequence alignment was performed using the Clustal Omega online tool. Alignment results were visualized via the ESPript 3.0 server, focusing on the conservation of the K591 site across different species.

Protein-Protein Interaction Network Prediction: Based on experimentally verified XRCC6 interaction partners listed in UniProt and combined with the STRING database, an initial XRCC6 PPI network was constructed. “Experimental evidence” and “database annotations” were set as high-confidence sources, with a minimum interaction score threshold of 0.700 to screen for high-confidence interacting proteins.

### 4.11 Breast cancer cell xenograft animal model

All animal experiments complied with the regulations of the Institutional Animal Care and Use Committee. Nude mice (**Supplementary Table 5**) were randomly divided into two groups (n=6): the Control group (inoculated with MCF-7 cells stably transfected with the empty vector) and the K591R Mutant group (inoculated with MCF-7 cells stably expressing the XRCC6-K591R mutant). Log-phase cells were collected and resuspended in PBS. Each nude mouse received a subcutaneous injection of 100 μL of cell suspension into the left axilla. Specifically, two days prior to tumor inoculation, the mice were subcutaneously injected with 4 µL of estradiol valerate (10 mg/ml). For the inoculation process, MCF-7 cells were suspended in 100 µL of DMEM, thoroughly mixed with 20 µL of estradiol valerate, and then subcutaneously injected into the right forelimb of each nude mouse. Following the cell injection, the mice received additional estradiol valerate injections on days 2, 5, 9, 16, and 21 to maintain tumor growth. On day 21 post-inoculation, all mice were sacrificed by cervical dislocation. Subcutaneous tumors were surgically excised intact, photographed, and weighed.

### 4.12 Human breast cancer samples

Human breast cancer tissues and matched adjacent non-tumor tissues were obtained from Xinxiang Central Hospital, with the study protocol approved by the Ethics Committee of Xinxiang Central Hospital. A portion of the tissue samples was processed into paraffin sections for immunohistochemical (IHC) staining. The remaining tissues were utilized for protein extraction and subsequent Western blot analysis.

### 4.13 Statistical analysis

Cell counts for invasion and colony formation assays were determined using ImageJ software with defined particle size parameters. For immunofluorescence images, protein levels were quantified by measuring fluorescence intensity in ImageJ. All statistical analyses were performed using GraphPad Prism 8. Data are expressed as mean ± standard deviation (SD). Statistical significance between two groups was evaluated using an unpaired, two-tailed Student’s t-test, A *P* < 0.05 was considered to indicate a statistically significant difference.

## Data Availability

The original contributions presented in the study are included in the article/**Supplementary Material**, further inquiries can be directed to the corresponding author/s.
