# Supplementary material for: SIRT7-mediated deacetylation of XRCC6 at lysine 591 drives breast cancer progression
Source: Front Oncol. 2026 May 8;16:1806267. doi: 10.3389/fonc.2026.1806267 (PMC13194032; doi:10.3389/fonc.2026.1806267)
Supplement: Supplementary file 1 [file Table1.docx]

**Supplement tables**

**Table 1. Clinical Specimen Information**

| **Sample Type** | **Source / Patient Profile** |
| --- | --- |
| Breast Cancer Tissue | **Patient Demographics & Clinicopathological Characteristics:** • Age: 36–72 years (median: 54) • Histological type: Invasive carcinoma (majority), including ductal, lobular, mucinous, medullary features • Disease stage: 0–IIB (majority I–IIA) • Molecular subtype: Luminal B (9), Luminal A (6), TNBC (3), HER2-positive (2) • HER2 status: 0 to 3+ (including FISH negative cases) • Ki67 range: ~5% to ~90%  **Detailed Patient Information:**   \| **Case ID** \| **Age** \| **Histology** \| **Stage** \| **Molecular Subtype** \| **HER2** \| **Ki67** \| \| --- \| --- \| --- \| --- \| --- \| --- \| --- \| \| 2553464201 \| 64 \| Right breast invasive carcinoma \| Ⅰ (T1cN0M0) \| Luminal B \| 2+ \| - \| \| 2508385301 \| 54 \| Right breast invasive carcinoma \| Post-neoadjuvant 0 (ycT0N0M0) \| HER2-positive \| 3+ \| - \| \| 2508404301 \| 70 \| Left breast invasive carcinoma with medullary features \| ⅡB (pT2N1M0) \| Triple-negative \| 2+ \| ~90% \| \| 2553458801 \| 62 \| Left breast invasive carcinoma \| Ⅰ (T2N0M0) \| Luminal B \| 2+ \| ~30% \| \| 2508361501 \| 47 \| Left breast invasive carcinoma \| Post-neoadjuvant ⅡA (ycT2N1M0) \| Luminal B \| 1+ \| ~5% \| \| 2508413501 \| 40 \| Left breast invasive carcinoma \| ⅡB (T3N0M0) \| Luminal A \| 1+ \| ~20% \| \| 2508623101 \| 72 \| Left breast invasive carcinoma \| Ⅰ (T2N0M0) \| Luminal A \| 1+ \| ~30% \| \| 2508663201 \| 53 \| Left breast invasive carcinoma \| ⅡA (pT2N0M0) \| Triple-negative \| 0 \| ~60% \| \| 2553589301 \| 45 \| Left breast invasive lobular carcinoma \| 0 (TisN0M0) \| Luminal B \| 2+ \| ~20% \| \| 2553583701 \| 65 \| Right breast invasive carcinoma \| ⅠA (pT1bN0M0) \| Luminal B \| 2+ \| ~5% \| \| 2508822001 \| 36 \| Left breast invasive tubulocarcinoma + DCIS \| ⅠA (pT1bN0M0) \| Luminal A \| 2+ (FISH-) \| ~5% \| \| 2508038601 \| 65 \| Left breast invasive carcinoma \| Post-neoadjuvant ⅡB (ypT2N2M0) \| Luminal A \| 2+ \| ~5% \| \| 2508101201 \| 48 \| Right breast invasive carcinoma \| ⅠA (pT1cN0M0) \| Luminal B \| 3+ \| ~5% \| \| 2553334801 \| 65 \| Left breast invasive carcinoma \| ⅡA (pT1cN1M0) \| Luminal B \| 2+ \| ~25% \| \| 2553254901 \| 66 \| Right breast invasive carcinoma \| Post-neoadjuvant ⅡA (ypT1-2N1M0) \| Triple-negative \| 2+ (FISH-) \| ~60% \| \| 2508203001 \| 45 \| Left breast invasive carcinoma + DCIS \| ⅡA (pT1cN1M0) \| HER2-positive \| 3+ \| ~50% \| \| 2553274201 \| 56 \| Right breast invasive carcinoma \| ⅡA (pT2N0M0) \| Luminal B \| 2+ \| ~75% \| \| 2553358201 \| 37 \| Left breast mucinous carcinoma \| ⅠA (pT1cN0M0) \| Luminal A \| - \| ~10% \| \| 2508065301 \| 47 \| Left breast invasive carcinoma + DCIS \| ⅡA (pT1cN1M0) \| Luminal B \| - \| ~30% \| \| 2553401201 \| 37 \| Right breast invasive carcinoma \| Ⅰa (pT1N0M0) \| Luminal A \| 2(0) \| ~7% \| |
| Adjacent Normal Tissue (>2 cm from tumor) | Paired non-tumor tissue from the same patients listed above. |
| **Sample Type** | **Source** |
| Breast Cancer Tissue | Patients undergoing surgery (no neoadjuvant therapy).Immediately frozen in liquid nitrogen after resection |
| Adjacent Normal Tissue (>2 cm from tumor) | Same as above |

**Table 2. Cells and Plasmids**

| **Name** | **Type/Description** | **Source** |
| --- | --- | --- |
| MCF-7 | breast cancer cell line | ATCC |
| T47D | breast cancer cell line | ATCC |
| pCDH-CMV-XRCC6-Flag | XRCC6 wild-type overexpression plasmid (C-terminal Flag tag) | Constructed by Hanbio |
| pLKO.1-shXRCC6 | XRCC6-specific shRNA knockdown plasmid | Constructed by Hanbio |
| pCDH-CMV-XRCC6-K591Q-His | XRCC6 K591Q point mutation plasmid | Yongyu BioTech |
| pCDH-CMV-XRCC6-K591R-Flag | XRCC6 K591R point mutation plasmid | Yongyu BioTech |
| pCDH-CMV-MCS-Flag (Empty vector) | Overexpression control plasmid | Hanbio |
| pLKO.1-scramble | Knockdown control plasmid | Hanbio |

**Table 3. Main Reagents**

| **Reagent Name** | **Manufacturer** | **Catalog Number** | **Application/Notes** |
| --- | --- | --- | --- |
| DMEM High Glucose Medium | Cytiva (USA) | SH30022.01 | Cell culture |
| Fetal Bovine Serum | BIOIND (Israel) | 04-001-01A | Cell culture |
| Penicillin-Streptomycin Solution | Beyotime (China) | C0222 | Cell culture |
| Trypsin-EDTA Solution | Beyotime (China) | C0201 | Cell digestion |
| Puromycin | MedChemExpress (USA) | HY-B1743A | Stable cell line selection |
| Matrigel Matrix | Corning (USA) | 356234 | Transwell invasion assay |
| Cycloheximide (CHX) | MedChemExpress (USA) | HY-12320 | Protein stability assay (100 μg/mL) |
| NAM (SIRT7 inhibitor) | Sigma (USA) | N0636 | Deacetylation inhibition (5 mM) |
| C646 (EP300 inhibitor) | TargetMol (USA) | T6133 | Acetylation inhibition (10 μM) |
| Oxaliplatin | Meilunbio (China) | MB1205 | Platinum drug treatment |
| Paraformaldehyde | Beyotime (China) | P0099 | Cell fixation |
| Crystal Violet Stain | Beyotime (China) | C0121 | Cell staining |
| RIPA Lysis Buffer | Beyotime (China) | P0013B | Total protein extraction |
| Protease Inhibitor Cocktail | Beyotime (China) | P1005 | Protein extraction |
| Deacetylase Inhibitor | MedChemExpress (USA) | HY-193021/193022 | Acetylated protein protection |
| BCA Protein Assay Kit | Beyotime (China) | P0010 | Protein quantification |
| SDS-PAGE Gel Preparation Kit | Beyotime (China) | P0012A | Western blot |
| ECL Chemiluminescent Substrate | Beyotime (China) | P0018 | Western blot detection |
| TRIzol Total RNA Extraction Reagent | Thermo Fisher (USA) | 15596026 | RNA extraction |
| Reverse Transcription Kit | Accurate Biology (China) | AG11728 | cDNA synthesis |
| SYBR Green qPCR Master Mix | Applied Biosystems (USA) | A25743 | Real-time quantitative PCR |
| CCK-8 Cell Proliferation Assay Kit | MedChemExpress (USA) | HY-K0301 | Cell proliferation assay |
| Annexin V-FITC/PI Apoptosis Detection Kit | KeyGEN BioTECH (China) | KGA105-KGA108 | Cell apoptosis assay |

**Table 4. Antibodies**

| **Antibody Name** | **Host** | **Manufacturer** | **Catalog Number** | **Application (Dilution Ratio)** |
| --- | --- | --- | --- | --- |
| Rabbit anti-human XRCC6 Antibody | Rabbit | Abcam (UK) | ab92450 | WB (1:1000) |
| Mouse anti-Flag Tag Antibody | Mouse | Sigma (USA) | F1804 | WB (1:1000), IP (1-2 μg) |
| Rabbit anti-Acetylated Lysine (Ac-K) Antibody | Rabbit | CST (USA) | 9441 | WB (1:1000), IP (1:50) |
| Rabbit anti-PARP1 Antibody | Rabbit | Abcam (UK) | ab32138 | WB (1:1000) |
| Rabbit anti-EP300 Antibody | Rabbit | Abcam (UK) | ab275378 | WB (1:1000) |
| Rabbit anti-SIRT7 Antibody | Rabbit | Abcam (UK) | ab259968 | WB (1:1000) |
| Mouse anti-β-actin Antibody | Mouse | Affinity (China) | AF7018 | WB (1:5000) |
| HRP-conjugated Goat anti-Rabbit IgG | - | Beyotime (China) | A0208 | WB (1:5000) |
| HRP-conjugated Goat anti-Mouse IgG | - | Beyotime (China) | A0216 | WB (1:5000) |

**Table 5. Experimental Animals**

| **Animal Strain** | **Gender** | **Age** | **Source** | **Housing Conditions** | **Purpose** |
| --- | --- | --- | --- | --- | --- |
| BALB/c Nude Mice | Female | 4-6 weeks | Vital River Laboratory Animal Technology (China) | SPF environment | Subcutaneous xenograft tumor model |

**Table 6. PCR primers**

| Primers for qPCR analysis | | |
| --- | --- | --- |
| GAPDH-1 | Forward | TCACCATCTTCCAGGAGCGA |
|  | Reverse | TGGACTCCACGACGTACTCA |
| GAPDH-2 | Forward | ACCATCTTCCAGGAGCGAGA |
|  | Reverse | GACTCCACGACGTACTCAGC |
| XRCC6-1 | Forward | TTGGCTGTGGTGTTCTATGGT |
|  | Reverse | AGAATTCGTTTTGCACCTGGAT |
| XRCC6-2 | Forward | GTTCTATGGTACCGAGAAAGACA |
|  | Reverse | TCTAGAATTCGTTTTGCACCTGG |
