# Supplementary material for: SIRT7-mediated deacetylation of XRCC6 at lysine 591 drives breast cancer progression
Source: Front Oncol. 2026 May 8;16:1806267. doi: 10.3389/fonc.2026.1806267 (PMC13194032; doi:10.3389/fonc.2026.1806267)
Supplement: Supplementary file 2 [file DataSheet1.docx]

**Supplementary Figure**

**
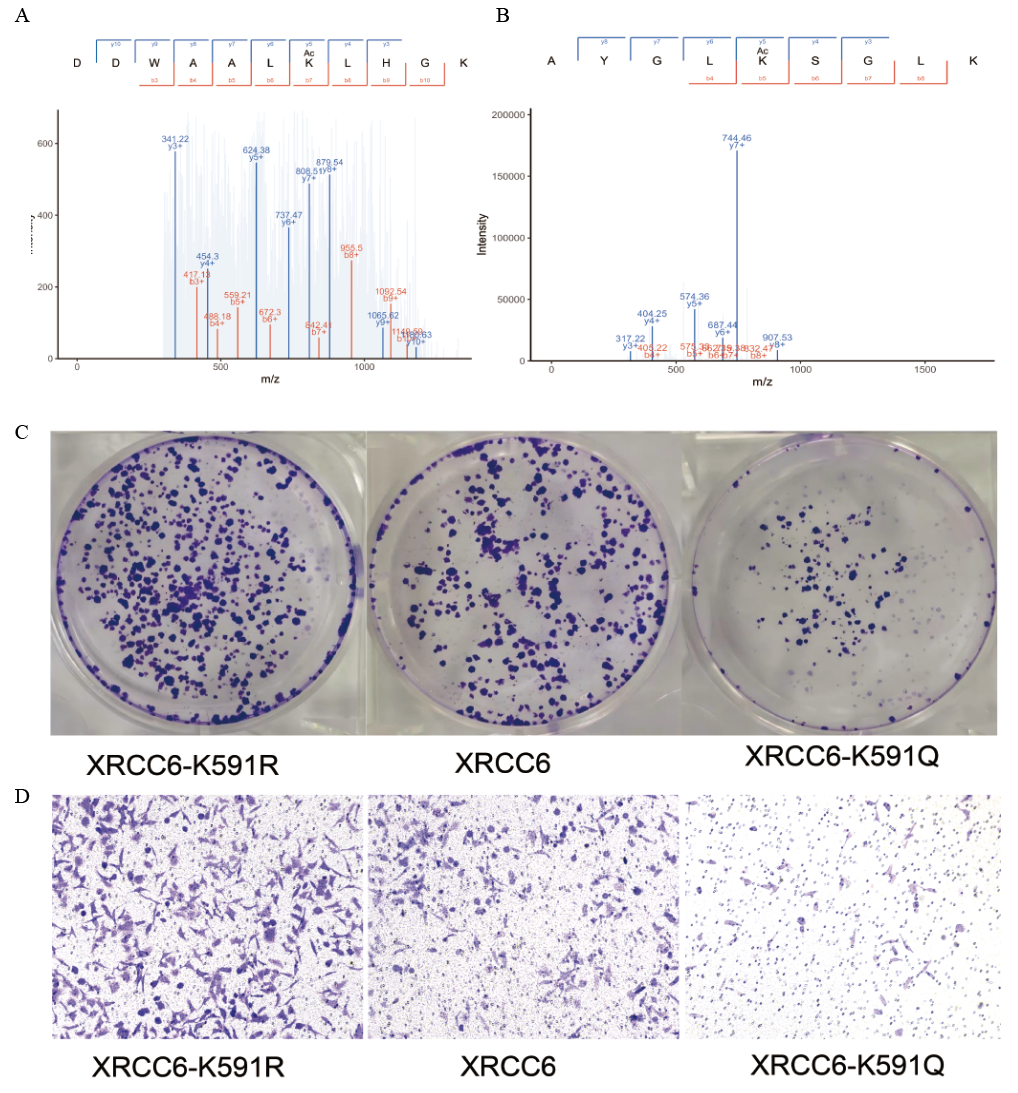
**

**Figure 1.**

**A.** Representative mass spectrum of Sirt7 peptides. **B.** Mass spectrum identifying the acetylation site at K591 of XRCC6. **C** and **D**. Transwell, colony formation, and wound healing assays demonstrated that the non-acetylatable K591R mutant enhanced the invasion, colony formation, and migration capabilities of T47D cells, whereas the acetylation-mimetic K591Q mutant displayed the opposite effect.
